# Exploratory Characterization of Artisanal Royal Jelly Produced by *Apis mellifera siciliana* Using ^1^H-NMR Metabolomics and NIR Spectral Fingerprinting

**DOI:** 10.3390/foods15173101

**Published:** 2026-09-01

**Authors:** Cesare Ravagli, Federica Pasini, Valentina Antonioni, Davide Cirrito, Chenglin Zhu, Maria Fiorenza Caboni, Luca Laghi

**Affiliations:** 1 Department of Agricultural and Food Sciences, University of Bologna, Piazza Goidanich 60, 47521 Cesena, FC, Italy; cesare.ravagli2@unibo.it (C.R.); federica.pasini5@unibo.it (F.P.); valentina.antonioni@unibo.it (V.A.); davide.cirrito@studio.unibo.it (D.C.); maria.caboni@unibo.it (M.F.C.); 2 Interdepartmental Centre of Industrial Agri-Food Research (CIRI Agroalimentare), University of Bologna, Via Quinto Bucci 336, 47521 Cesena, FC, Italy; 3 College of Pharmacy and Food, Southwest Minzu University, Chengdu 610041, China; chenglin.zhu@swun.edu.cn

**Keywords:** royal jelly, metabolomics, ^1^H NMR spectroscopy, NIR spectroscopy, *Apis mellifera siciliana*

## Abstract

Italian production of royal jelly (RJ) is modest compared to imports, as domestic producers face much higher production costs than their foreign counterparts. To mitigate the competitive advantage of imported products, Italian producers could adopt marketing strategies emphasizing the local origin of their royal jelly. An especially interesting opportunity for Sicilian producers lies in RJ obtained from the *siciliana* spp. of *Apis mellifera*, commonly known as the black honeybee, rather than from the widespread *A. mellifera ligustica*. This study employed ^1^H-NMR and NIR spectroscopies to identify key metabolomic traits of artisanal royal jelly (n = 5) produced by *A. m. siciliana* in order to differentiate it from mass-produced (n = 5) and artisanal samples from *A. m. ligustica* (n = 6). Commercially available samples, whose market price was at least five times lower than Italian artisanal products, were used as references to represent mass-produced RJ from *A. mellifera ligustica*. To validate the suitability of the discriminant metabolites or wavelengths as potential biomarkers, an unsupervised multivariate model was built on these features and subsequently applied to additional artisanal samples produced by *A. m. ligustica* of known Italian origin. Among the 36 metabolites identified by ^1^H-NMR, seven showed statistically significant differences between *A. m. siciliana* and mass-produced samples. Notably, glucose, melibiose and UDP-glucose were more abundant in commercial samples. Artisanal samples from *A. m. ligustica* exhibited intermediate features between those from *A. m. siciliana* and mass-produced samples from *A. m. ligustica*, while specific differences between the two artisanal groups were also observed. NIR spectroscopy further confirmed distinct profiles between artisanal *A. m. siciliana* and mass-produced samples from *A. m. ligustica*, with Italian artisanal samples from *A. m. ligustica* showing greater similarities to the former.

## 1. Introduction

Royal jelly (RJ) is produced by worker honeybees to feed the queen throughout her life and the honeybee larvae for the first few days after hatching [1]. The species employed for RJ production all around the world is *Apis mellifera*.

The main components of RJ are water (60–70%), proteins (9–18%), carbohydrates (7–18%), lipids (3–8%), and B-complex vitamins. Its commercial interest is related to compounds with antimicrobial and antioxidant properties. A prominent role in this respect is played by 10-hydroxy-2-decenoic acid (10-HDA); because no natural product but RJ is reported to contain this fatty acid, its presence is the main criterion in RJ’s authenticity control [2]. RJ molecules are synthesized in honeybees’ glands, with hypopharyngeal glands (HPGs) mainly responsible for proteins synthesis and mandibular glands (MGs) predominantly secreting lipid constituents, mainly from pollen components [3].

The amount of RJ produced per colony and its composition depend on several factors, including the honeybee subspecies and strain, climatic conditions, colony strength, and pollen availability [4,5,6,7,8]. Interestingly, being a glandular secretion, both the amount per bee and the composition depend only marginally on the flowers’ species providing the pollen [9,10].

Its antimicrobial, antioxidant, anti-inflammatory, and immunomodulatory properties have drawn significant commercial interest, especially within the nutraceutical and cosmetic industries, so that its global market was estimated at 99.15 million USD in 2024 [11]. In 1984, annual worldwide production of RJ was 700 metric tons, with 60% from China. In 2011, China increased production to 3500 tons per year [11], capturing 95% of the global market [12,13]. The success of Chinese production is mainly due to the import at the end of the 1800s of the subspecies *Apis mellifera ligustica*, native to the Italian region Liguria. Its nearly exclusive use for RJ production, coupled with the subsequent development of strains selectively bred for enhanced royal jelly production (HRJB) [11], allowed beekeepers to increase RJ’s yield from the 300 g per colony per year that was obtained at the beginning of *Apis mellifera ligustica*’s introduction in China to the 5 kg obtained nowadays.

The main driver of RJ’s high yield granted by these strains is that they organize in large colonies. Here, each nurse bee is not forced to produce royal jelly to its maximum capacity, making the individual bee, and therefore the colony, longer-lived. In addition, the production per nurse bee does not scale linearly with the size of the colony. This can be deduced by noting that when there are many grafted queen cells, the quantity of royal jelly per cell decreases only marginally [10]. Increases in royal jelly production have been also granted by specific hive management techniques and by tools such as plastic queen cups.

Compared to China, Italian RJ production is modest and has been put under pressure in recent years by the decrease in the net population of bees, triggered by climate changes and by differences in production costs compared to the Chinese counterpart. The magnitude of this difference can be appreciated considering that, in 2014, China exported RJ at 53 USD per kg [11], while the Italian consortium COPAIT (Associazione per la produzione e la valorizzazione della pappa reale fresca italiana—Association for the production and promotion of fresh Italian royal jelly) reports a wholesale price for the Italian certified product approximately 10 times higher. As a confirmation of this difference, at the time of writing this work, specialized Italian websites sell RJ from unspecified origins at 0.4 euros per gram, while RJ certified as Italian by COPAIT goes for 2.5 euros per gram [14]. Consequently, domestic RJ production accounts for less than 10% of Italian demand, with the remainder being met by imports.

To help offset the competitive advantage of mass-produced, imported products, Italian producers could adopt marketing strategies revolving around the local origin of their RJ, appealing especially to higher-income consumers [15]. A particularly interesting possibility, from this point of view, is represented by RJ produced by the *siciliana* subspecies of *Apis mellifera*, commonly known as the black honeybee, instead of the ubiquitous *ligustica.* The *siciliana* subspecies derives from African lineages, whereas *ligustica* derives from European lineages [16]. Genetically pure colonies have been conserved on the Sicilian Island of Ustica and today in a restricted area of west Sicily, where eight beekeepers produce the only honey entirely made by the black honeybee. The black honeybee differs from *ligustica* in morphologic characteristics, with a darker abdomen, yellow down, and smaller wings [17]. More importantly, the black honeybee also differs from *A. m. ligustica* in several physiological traits, having a better resistance to drought, high temperatures, and temperature fluctuations, as well as to varroa and related viruses [18]. Given the very restricted diffusion of the black honeybee, only a few scientific works have studied their products, and all of them have been devoted to honey. Notably, those works highlighted chemical characteristics and antimicrobial properties [18,19,20] endowing them with outstanding nutraceutical properties, including high antioxidant capacity [21]. At the time of writing the present work, no study has been devoted to the black honeybee’s RJ.

In the quest for the identification of peculiarities of food products with potential consequences for consumer’s appreciation, metabolomics approaches have been found perfectly tailored. Limited to bees’ products, Li et al. [22] applied untargeted metabolomics to assess how floral and geographical origins determined the composition of honey. In 2015, Mannina et al. identified compounds in honey produced by the Sicilian black honeybee that distinguished sulla honey from dill honey [20]. Li et al. [3] were able to distinguish by metabolomics the effects of bee artificial diets on the composition of RJ.

Among the analytical platforms typically employed for metabolomics investigations, proton nuclear magnetic resonance (^1^H-NMR) has earned a prominent role due to the very high reproducibility of the results granted through minimal sample preparation. Indeed, Mazzei et al. [23] obtained interesting results about the overall quality of the RJ, but also in terms of identification of fraudulent procedures when comparing the concentration of target molecules, like lysine and citrate, between artisanal and commercial samples obtained from the Asiatic market. Near-infrared (NIR) spectroscopy provides complementary information on the profiles of sugars, proteins, fats, and fibers with minimal sample preparation, making it well suited to complement ^1^H-NMR analysis. Focusing on honeybee products, Ferreiro-González et al. [24] were able not only to spot, but even to quantify rapidly, honey adulterated with high fructose corn syrup.

This study aimed to identify key metabolomic traits of artisanal RJ produced by *siciliana* spp. honeybees. Commercially available samples, whose final price was at least five times lower than Italian artisanal products, were used as references to represent mass-produced products from *A. mellifera ligustica*. To confirm the reasonableness of using the molecules emerging from this comparison as differentiating biomarkers, an unsupervised multivariate model was built on them, which was then used to characterize additional samples produced by *ligustica* honeybees and of known artisanal Italian origin. These samples were Sicilian and non-Sicilian, collected in different months. This made them as descriptive as possible of the bee species and preparation method, while minimizing the potential influence of pollen botanical origin and the regional climate conditions.

## 2. Materials and Methods

### 2.1. Samples

The study included 16 royal jelly (RJ) samples marketed as fresh, which were either purchased commercially or obtained directly from producers, as detailed in Table 1. The samples were divided into 3 main categories: 5 commercial samples (C), 6 artisanal samples obtained from bees of the *Apis mellifera ligustica* species (O) and 5 artisanal samples obtained from bees of the *Apis mellifera siciliana* species (S). The artisanal samples were produced without the use of external nutritional supplements by the beekeeper. To stimulate the initiation of queen-rearing processes, selected beehives were induced into a state of orphanhood by removing the queen from the colony. Subsequently, artificial queen cups were inserted into the hives to facilitate a controlled production. Young worker bee larvae (≤36 h old) were transferred into the artificial cups using a specialized grafting spoon (Anel Honey Park, Thessaloniki, Greece). Larvae of *Apis mellifera ligustica* were used for the O samples, whereas larvae of *Apis mellifera siciliana* were used for the S samples. In all cases, the larvae originated from colonies maintained by the same producer to ensure lineage purity. Following grafting, the larvae were reared for 5 days to allow maximal accumulation of royal jelly within the queen cups while preventing progression to advanced developmental stages. After this period, all artificial cups were collected, uncapped with a knife to remove the wax covering, and the royal jelly was extracted and filtered using a harvesting pump (AN65400, Anel Honey Park, Thessaloniki, Greece). The mean yield was approximately 10 g of royal jelly per 30 processed cups. All royal jelly samples were subsequently packaged in 10 g containers and stored at 4 °C to comply with commercial handling and preservation requirements of products to be marketed as fresh.

### 2.2. Moisture Content

The moisture content of the samples was measured following the protocol reported in ISO 12824:2016 [25]; 1 g of sample was weighed in a ceramic crucible and heated at 55 °C for 16 h in a thermostatic oven (PID system, type M 150—VF, Instruments s.r.l., Bernareggio, MI, Italy). After treatment, the samples were weighed again, and the moisture content was calculated.

### 2.3. NIR Analysis

The samples were analyzed using a BUCHI NIRFlex Solids N-500 instrument (BUCHI, Cornaredo, Italy), equipped with an XL Add-On accessory designed to reduce the sample surface area required for analysis. The instrument operated under reflectance conditions, utilizing an external white reference. For each sample, 7 to 10 g of RJ were employed to cover completely the analytical lens, and a homogeneous distribution was obtained by vortex mixing. NIR spectra were acquired with a 1 mm optical path length and recorded by an average of 8 scans of each sample in a range from 10,000 to 4000 cm^−1^ (1000–2500 nm), with a resolution of 4 cm^−1^. Each NIR spectrum was finally adjusted by moisture content to reduce the influence of the broad O–H absorption bands at approximately 6900 and 5150 cm^−1^.

### 2.4. ^1^H-NMR

For ^1^H-NMR analysis, approximately 50 mg of each sample was added to 1 mL of deionized water, vortex-mixed for 5 min and then centrifuged at 18,630× *g* for 10 min at 4 °C. A 0.70 mL aliquot of supernatant was mixed with 0.10 mL of 10 mM 3-(trimethylsilyl) propionic-2,2,3,3-d_4_ acid sodium salt (TSP) in D_2_O as a chemical-shift reference (δ −0.017). After a further centrifugation at the above conditions, 0.65 mL of supernatant was transferred to an NMR tube for analysis.

To register ^1^H-NMR spectra, an AVANCE III spectrometer (Bruker, Milan, Italy) was employed, operating at 600.13 MHz and equipped with the software Topspin (ver. 3.5). ^1^H-NMR spectra were acquired at 298 K using a CPMG pulse sequence with suppression of the solvent signal and the use of the following parameters: size of fid 32 k, number of scans 16, number of dummy scans 16, spectral width 12 ppm, acquisition time 2.28 s, delay d1 5 s. NMR spectra were baseline- and phase-adjusted in Topspin.

For the characterization and quantification of the molecules, signals assignment was performed in Chenomx software (Chenomx Inc., Edmonton, AB, Canada, ver. 8.3) through comparison with Chenomx’s (ver. 10) and HMDB’s (release 2) databases and through comparison with the literature [23]. To allow readers to follow the assignment process, a Chenomx-formatted spectrum is provided as Supplementary Materials, overlaid with spectra of pure reference compounds. The probabilistic quotient normalization procedure (PQN) [26] was employed to evidence the most representative sample in terms of solutes concentration. Absolute quantification of the metabolites was performed on each sample by using TSP as an internal standard. Spectra from all the samples were then adjusted towards the reference by PQN to compensate for differences in the content of water and insoluble solids. The concentration of each molecule was calculated from the area of one of its signals, measured by global spectra deconvolution implemented in MestReNova software (Mestrelab research S.L., Santiago De Compostela, Spain—ver. 14.2.0-26256). This was done after applying a line broadening of 0.3 and a baseline adjustment by the Whittaker Smoother procedure and by considering an LOQ equal to 5.

### 2.5. Statistical Analysis

Statistical analysis was performed in the R computational language (ver 4.0.2). Comparisons between two groups were then conducted by the Wilcoxon test. Adjustments of *p*-values for the false discovery rate (FDR) was performed using the Benjamini–Hochberg procedure. Trends characterizing the samples were identified using robust principal component analysis (rPCA) models, based on centered and scaled metabolomics or NIR data selected through univariate analysis. The PcaHubert algorithm from the “rrcov” package (ver. 1.5-5) was employed to ensure robustness [27]. This algorithm utilizes a two-step approach: initially, outlier samples are detected based on their distances, both along and orthogonal to the PCA plane; subsequently, the optimal number of principal components (PCs) is determined. Each rPCA model is described through two key visualizations: a scoreplot and a Pearson correlation plot. The scoreplot represents the projection of the samples in the PC space, revealing the underlying data structure, while the correlation plot reveals the importance of each original variable to the components of the model. Correlations between metabolomics data and NIR spectra wavelengths were calculated using Pearson correlation.

## 3. Results

### 3.1. Metabolome

**Figure 1:**
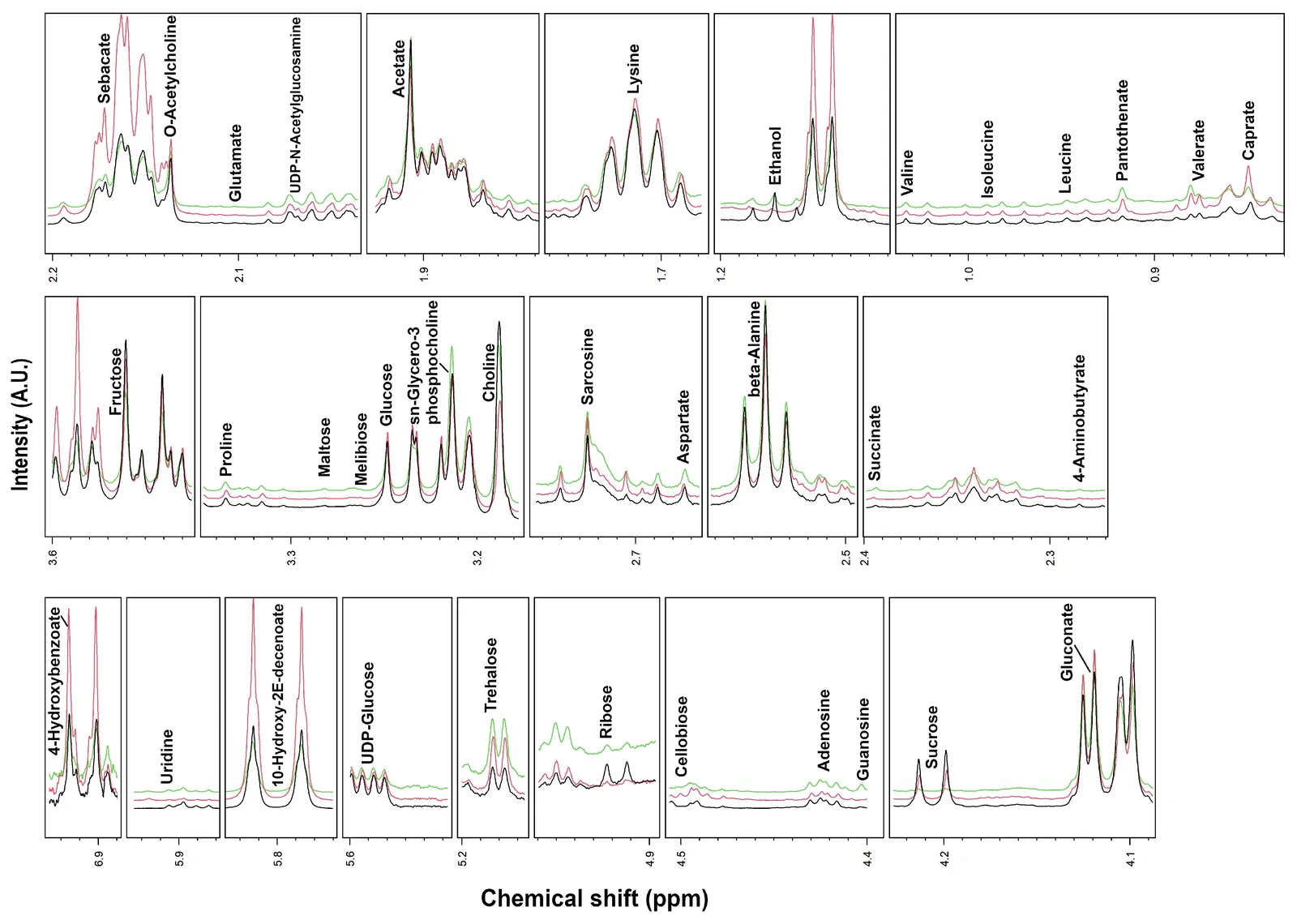
^1^H-NMR spectra representative of commercial (black), Italian *ligustica* (red) and *siciliana* (green) samples. Each portion has been individually scaled to ease visual inspection. The names of the molecules appear on the signals used for quantification.

^1^H-NMR allowed the quantification of 36 molecules in all the analyzed samples, as reported in Figure 1 and in the spectrum in Chenomx format provided as supplemental material. Among them, it is possible to notice amino acids or their derivatives (proline, valine, isoleucine, leucine, lysine, glutamate, aspartate, β-alanine, 4-aminobutyrate and sarcosine), carbohydrates or molecules related to their metabolism (glucose, fructose, sucrose, maltose, trehalose, ribose, cellobiose, melibiose, UDP-glucose), nucleosides and nucleotides (adenosine, guanosine, uridine), fatty acids and derivatives (10-Hydroxy-2E-decenoate, caprate, valerate, sebacate, acetate) and choline together with its related compounds sn-glycero-3-phosphocholine and O-acetylcholine.

As a preliminary step, we evaluated whether collecting samples from specific producers within fairly restricted areas (samples S and O), versus using mass-produced commercial samples likely originating from a much wider area (samples C), affected the overall variability of the groups. To this end, metabolite concentrations were centered and scaled, and intragroup distances were computed. No significant differences in dispersion were observed among the three groups.

### 3.2. Metabolome Features of RJ from Siciliana Honeybee Compared to Commercial Samples

**Figure 2:**
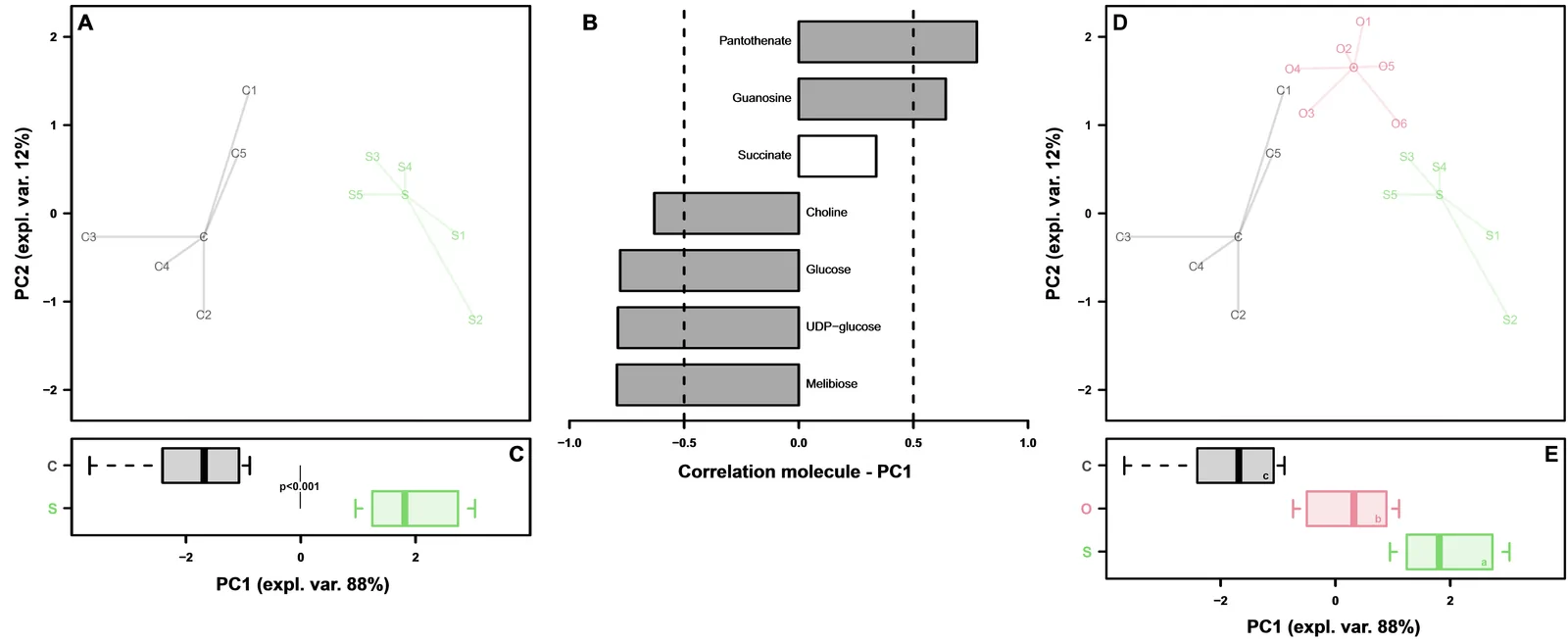
rPCA on the molecules quantified via ^1^H-NMR and found to be significantly different between samples C and S. (**A**) In the scoreplot, the letters C and S indicate the mean of the 2 groups analyzed. (**B**) Loading plot indicating the correlation between the concentration of the molecules and their importance in determining the PC 1. Dark grey indicates the significant correlations. (**C**) Boxplot summarizing the position along the PC 1 of the two groups of samples. (**D**,**E**) Scoreplot and boxplot corresponding to panels (**A**) and (**C**), respectively, with the addition of the predicted samples from group O. In panel (**E**), different lowercase letters highlight significant differences.

Seven of the 36 molecules quantified across all samples differed significantly between the S and C groups based on unadjusted *p*-values (Table 2 and Table S1 and Figure S1). After Benjamini–Hochberg correction, these differences were no longer statistically significant, although the molecules retained a trend toward differences between the groups (*p* < 0.1). The overall trend of these seven molecules was effectively captured by an rPCA model (Figure 2). In the scoreplot shown in panel A, commercial samples appear at negative PC 1 values and *siciliana* samples at positive values, significantly different from the former (*p* < 0.001). The loading plot in panel B indicates that the molecules that mostly contribute to this distribution are pantothenate and guanosine, more concentrated in S samples, and melibiose, UDP-glucose, glucose, and choline, more concentrated in commercial samples.

The overall trend of the seven metabolites captured by rPCA proved to be particularly suitable for observing how the samples of set O, artisanal from *ligustica* subspecies, compared with the other two sets of samples. To do so, the rPCA model was applied to the samples of group O. In Figure 2D, they appear projected in the scoreplot of Figure 2A, significantly separated along PC 1 from both the C and S samples, as highlighted by an ANOVA test followed by a Tukey HSD post hoc test (Figure 2E). Moreover, samples pertaining to group O were found to be equally distant from those of groups C and S.

### 3.3. Metabolome’s Comparison of Artisanal RJ from Siciliana and Ligustica Subspecies

The metabolomic features of RJ artisanal samples mainly linked to the subspecies of honeybee could be studied by comparing the samples of the groups S and O. Ten molecules differed significantly between the S and C groups based on unadjusted *p*-values (Table 3 and Table S2). Among them, nine showed significant differences also after Benjamini–Hochberg correction. Samples from *siciliana* subspecies showed lower concentrations of the fatty acids 10-hydroxy-2E-decenoate, sebacate, and valerate, together with three other molecules, while they had higher concentrations of adenosine, choline, guanosine, and uridine.

### 3.4. NIR Spectra Features Differentiating Artisanal siciliana Samples from Commercial Samples

**Figure 3:**
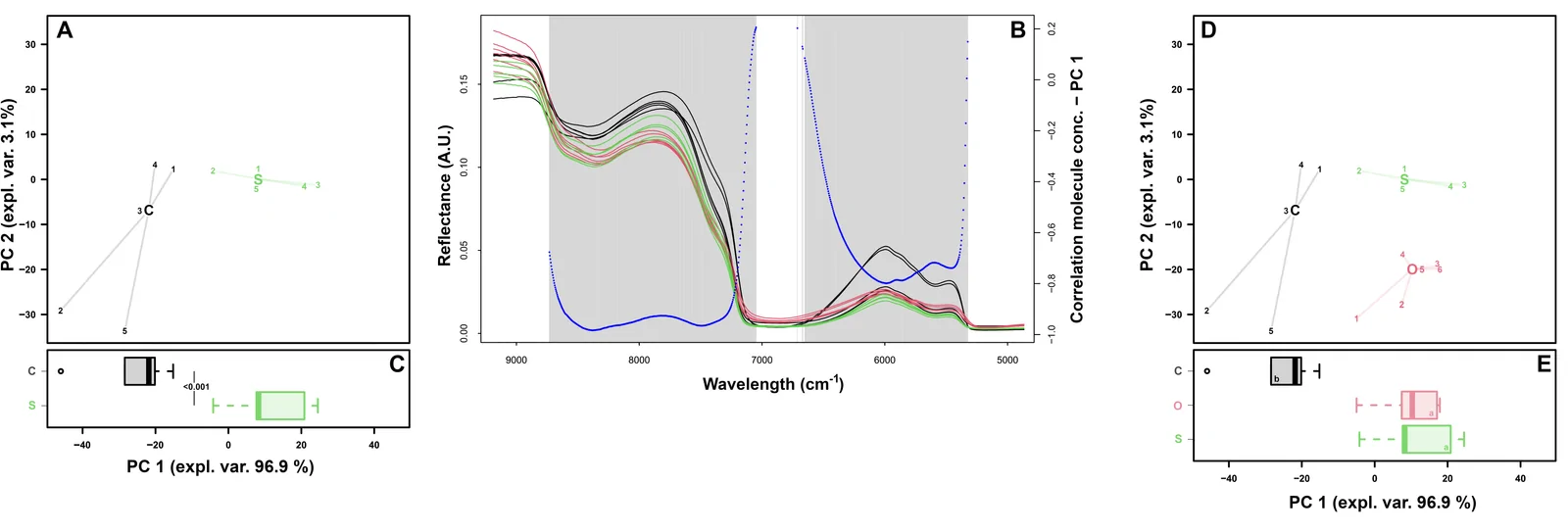
rPCA on wavelengths found to be significantly different between samples C and S. (**A**) In the scoreplot, the letters C and S indicate the mean of the 2 groups analyzed. (**B**) Black, red and green lines describe the NIR spectra registered on C, O and S samples, respectively. Wavelengths significantly different between samples C and S are highlighted by a gray background. The blue line quantifies the correlation between the wavelengths intensities and their importance in determining the PC 1. (**C**) Boxplot summarizing the position along the PC 1 of the two groups of samples. (**D**,**E**) Scoreplot and boxplot corresponding to panels (**A**) and (**C**), respectively, with the addition of the predicted samples from group O.

Similarly to what was done for the metabolomic data, a Wilcoxon test was employed to find the wavelengths at which the C and S samples showed significant differences (Figure 3B). On these samples, and only on the significantly different wavelengths, an rPCA model was calculated. In its scoreplot (Figure 3A), the commercial samples appear at negative PC 1 values and the S samples at positive values, significantly different from the former (*p* < 0.001).

The rPCA model so created was used to observe how samples O, artisanal from *ligustica* subspecies, compared with the other two sets of samples. This was done by applying the rPCA model itself to the samples from the group O. In Figure 3D, they appear projected in the previous scoreplot, significantly separated along PC 1 only from the C samples, as highlighted by an ANOVA test followed by a Tukey post hoc test.

### 3.5. Correlation Metabolome-NIR

**Figure 4:**
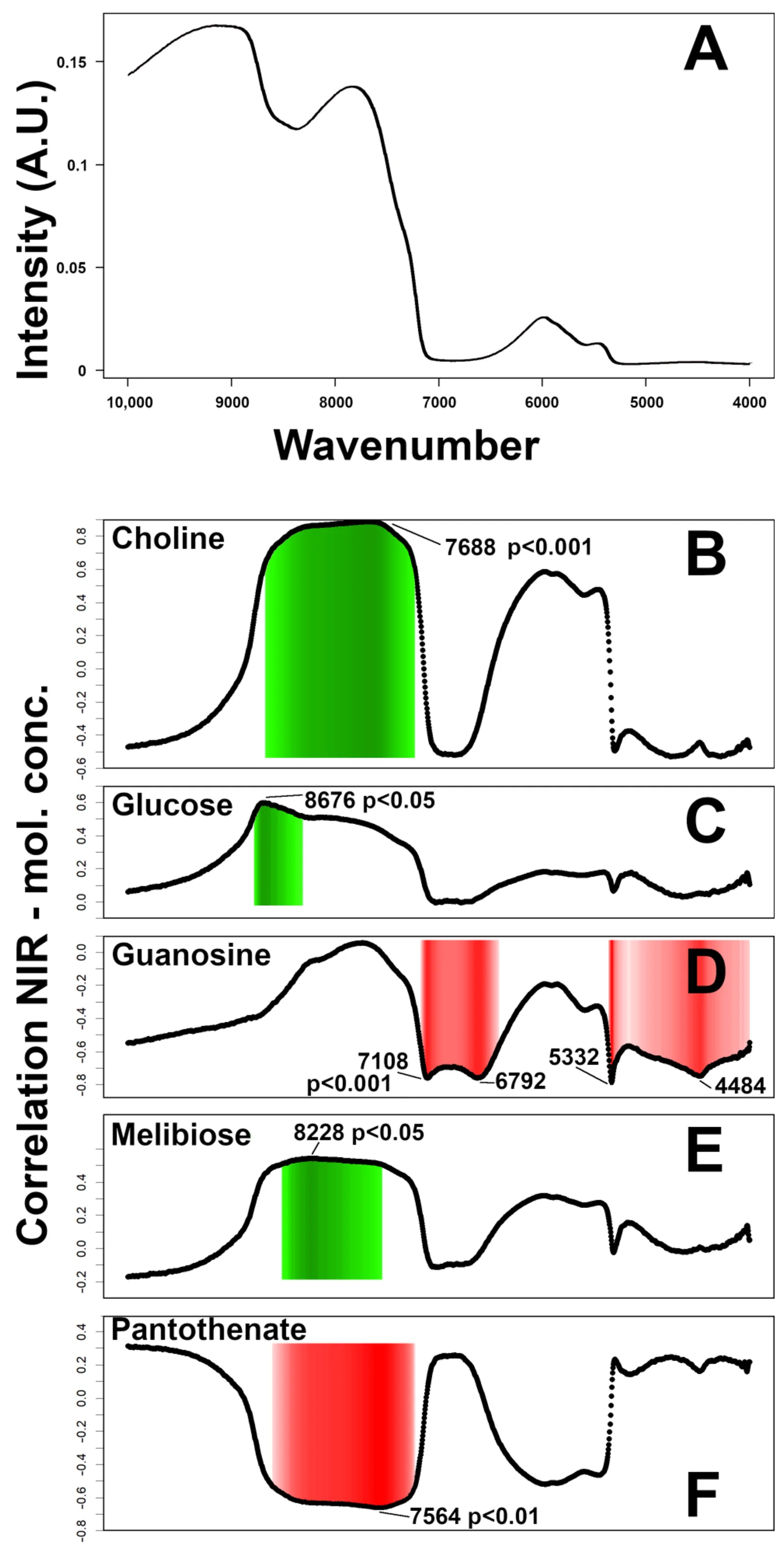
Pearson correlation between the NIR spectra points and the concentrations of the molecules evidenced by the rPCA model describing the metabolome. (**A**) Example NIR spectrum. (**B**–**F**) Black lines represent the r-values of the correlations. Green and red lines evidence direct or inverse relationships, with an intensity proportional to the *p*-value. Key wavenumbers are reported also numerically, together with the corresponding *p*-values.

To ease the interpretation of the information from NIR spectra, their wavelengths were correlated with the concentrations of the molecules that resulted in being significantly different between C and S samples and, at the same time, resulted in being significantly important in determining the distribution of the samples in the rPCA model of Figure 2. Figure 4 reports these correlations, except those with UDP-glucose, with an absolute value never higher than 0.5.

### 3.6. Moisture Content and Correlation with Metabolome

**Figure 5:**
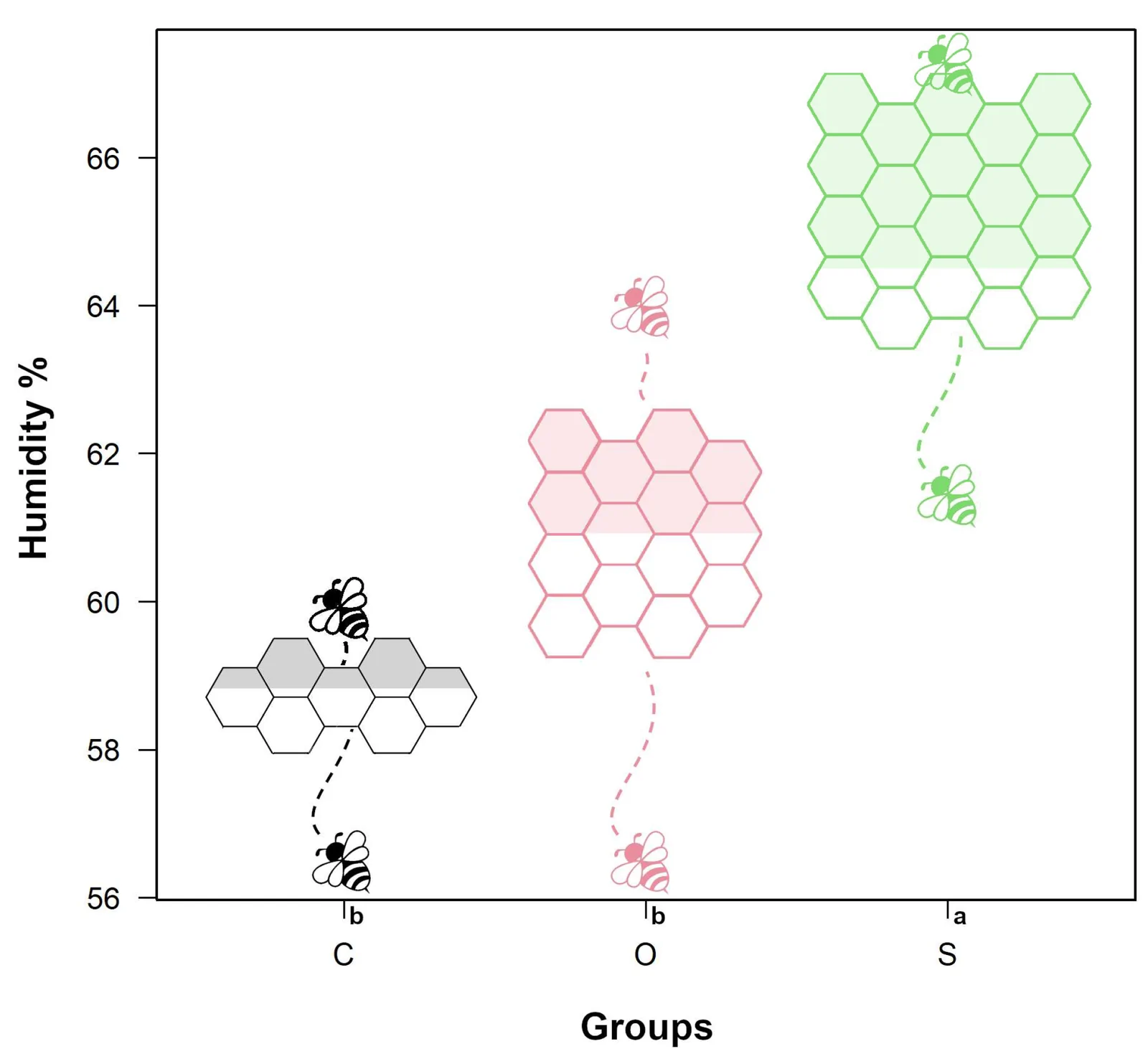
Boxplot of the moisture content for the three groups of samples. Different lowercase letters highlight significant differences (*p* < 0.05).

**Figure 6:**
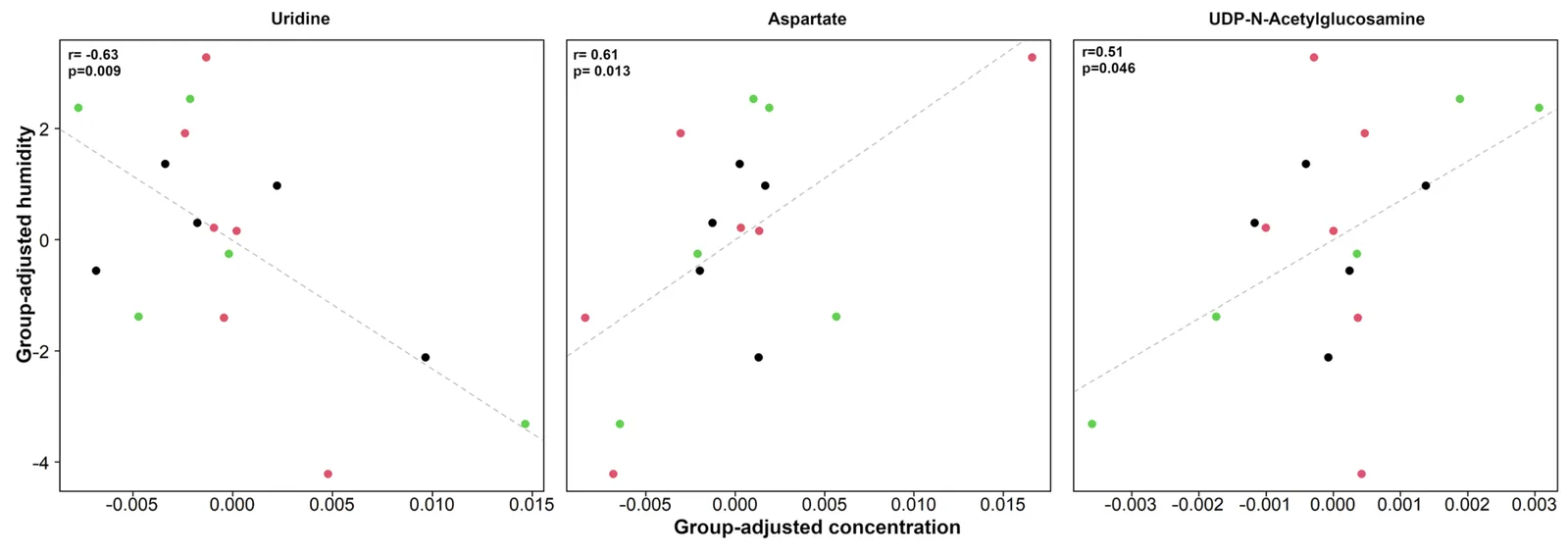
Pearson’s correlation between the group-adjusted humidity and metabolome. Only significant correlations are shown.

A peculiarity of the *siciliana* subspecies compared to *ligustica* is its adaptation to withstand draught conditions. To observe the potential consequences to the composition of the final product, we first evaluated the water content in the 16 samples analyzed (Figure 5). Indeed, samples from the S group had 64.75% of water, significantly higher than the 60.68% and the 58.51% of the O and C samples, respectively. Subsequently, we investigated if specific features of the metabolome could be linked to samples’ humidity, independently from the species of bee. To do so, from each molecule and from humidity values, the variance that could be ascribed to the bees’ species was removed, and then the Pearson’s correlation between the residuals was calculated. Three molecules showed a significant correlation, as reported in Figure 6.

## 4. Discussion

This work aimed at identifying peculiarities of artisanal RJ from the *siciliana* honeybee subspecies by comparing it with mass-produced or artisanal Italian samples from the *ligustica* subspecies. When artisanal RJ from the *siciliana* honeybee subspecies (S) was compared to mass-produced commercial samples (C), seven molecules appeared differently concentrated. Among them, the nucleoside guanosine was found more concentrated in S samples, potentially because this nucleoside could support larval biosynthetic demands. In line with this observation, higher levels of pantothenate, essential for fatty acid metabolism and energy production, could imply a RJ more geared towards supporting high metabolic rates. Similar considerations apply to succinate, an intermediate of the TCA cycle. Overall, higher concentrations of these three molecules might suggest an adaptation of the *siciliana* subspecies to harsher environments, favoring a RJ composition that is more resilient to spoilage and more oriented towards the growth and nurturing of the larvae. The sugars glucose and melibiose, more concentrated in C samples, could reflect environmental conditions, for example, more abundant sources of pollen, a common management practice to maximize RJ production [28].

A comparison between S and C products would benefit from the quantification of citrate. Mazzei et al. [23] reported higher citrate concentrations in Italian than in Chinese royal jelly, while Sari et al. [29] found that citric acid could help distinguish Italian from Brazilian products. Noteworthy, as can be observed in the Chenomx-formatted spectrum provided as Supplementary Materials, citrate could not be reliably identified in any of the analyzed samples because its diagnostic signals overlapped with the more intense signals of aspartate and 10-Hydroxy-2E-decenoate in the same spectral regions. This is particularly unfortunate, as Mazzei et al. quantified this molecule from NMR spectra obtained through a protocol comparable to ours, but with a spectrometer operating at lower magnetic fields.

It is very interesting to notice that in the reduced metabolomics space represented by the seven molecules differentiating samples from group S and C, samples from group O appeared with overall intermediate characteristics. This was particularly remarkable considering that samples from groups C and O were almost certainly generated from the same honeybee subspecies, the *ligustica*. The representation of the samples in this specific subspace seemed therefore to integrate differences connected to the production practices. This consideration, even if based on a very limited number of samples, is particularly appealing in the quest for strategies to enhance the intrinsic and perceived value of artisanal products.

NIR spectroscopy seemed very sensitive to the compositional differences between artisanal samples from the *siciliana* subspecies and commercial samples. Indeed, several wavelengths appeared differently excited by the two sets of samples, so that an rPCA model based on those wavelengths appeared to be very effective in capturing the overall differences between the samples. It is very interesting to notice that samples O projected on this reduced space could not be distinguished from S samples but appeared very different from C samples. This might suggest once more that production practices left traces in RJ samples’ overall molecular profile, confirming in turn the idea of combining ^1^H-NMR and NIR spectroscopy observations to distinguish artisanal from commercial samples. The subsequent search for correlations between molecules’ concentration and NIR wavelength intensities seems to reinforce the observations from both points of view. In fact, the wavelengths around 8200–8600 cm^−1^ are typical of carbohydrates’ C-H overtones, serving as a potential justification of the direct relationships between these bands and glucose and melibiose concentrations. Further support for this interpretation was provided by pantothenate, which was inversely associated with glucose and melibiose concentrations in the ^1^H-NMR data.

The water content of the analyzed RJ samples (Figure 5) might provide further insight into the adaptation of *A. m. siciliana* to dry climatic conditions. In dry periods, RJ from the *ligustica* subspecies is expected to show low water content because bees withdraw some of the water from RJ already produced [30]. In addition, high temperatures increase water evaporation noticeably. In disagreement with these general considerations, RJ from *siciliana* honeybees showed the highest hydration, even if meteorological data confirm that in June–July 2024, rainfall in Sicily was 72% less abundant compared to historical series [31].

Focusing specifically on the metabolomic differences between artisanal RJ produced by *A. m. siciliana* and *A. m. ligustica* (Table 3), lower concentrations of the fatty acids 10-HDA and sebacate were observed in S samples. However, it is unlikely that a single metabolic pathway underlies both trends. The presence of 10-HDA in RJ is linked to its biosynthesis from short-chain lipids, particularly acetate, deriving from pollen [32]. It is in fact well established that the abundance of pollen, or even its substitutes [3], leads to high concentrations of 10-HDA in the RJ. The hypothesis that *siciliana* bees may have experienced difficulty in finding pollen could be supported by the significantly low concentration of 4-hydroxybenzoate, also of exogenous origin [33].

Unlike 10-HDA, sebacate derives mainly from the catabolism of longer-chain fatty acids. Notably, Sari et al. [29] identified sebacate as a key discriminator of Indonesian RJ origins, with the lowest abundance observed in samples from the driest region. Overall, it seems that the drought that occurred in Sicily during the collection of samples from group S had no direct consequences for RJ’s humidity but did have consequences for RJ’s composition through the reduced pollen and nectar availability.

The intricate relation between water availability and RJ characteristics prompted us to investigate in greater detail any direct correlations between the metabolome and humidity of RJ. In this respect, it should be noted that RJ is synthesized by different glands of the nurse honeybees, even if each specializes in the secretion of different classes of molecules [34,35]. In humans, it is known that the increase in mandibular action caused by higher mastication leads to a different stimulation of the various saliva-producing glands, with consequences for the saliva composition [36]. Drought during RJ production may reduce pollen’s, and in turn RJ’s, humidity and may force nurse honeybees to increase mandibular action during production, with consequences for RJ’s composition. Our exploratory investigation suggests that there is indeed a correlation between hydration levels of RJ and the concentration of three molecules, namely, aspartate, UDP-acetylglucosamine and uridine. Interestingly, the three molecules do not appear in the list of those differentiating the samples of RJ produced by the *siciliana* subspecies honeybee from those produced by *ligustica*, both artisanal and commercial. This reinforces the idea that the differences evidenced in Table 2 between groups C and S and in Table 3 between groups C and O are mainly related to the subspecies of honeybee and to the different production management, and are only limitedly related to contingent weather conditions in the production areas.

## 5. Conclusions

The present study aimed to characterize, for the first time, the metabolome of artisanal royal jelly from *A. m. siciliana*. To do so, a comparison with artisanal and mass-produced commercial samples from *A. m. ligustica* was set up. The main limitation of this study lies in the latter comparison, as the details of the commercial samples could not be verified and had to be inferred from their market price and from the literature. A further limitation was represented by the low number of samples collected, limited by the extreme rarity of pure colonies of *A. m. siciliana*. Despite these limitations, the metabolome of royal jelly from *A. m. siciliana* appeared to be clearly distinguishable from the other products, using both ^1^H-NMR and NIR analyses. The differences did not translate into a nutritional superiority of artisanal royal jelly from *A. m. siciliana*, as evidenced by the low concentration of the fatty acid 10-HDA in the specific samples analyzed. Anyway, the peculiarities highlighted may represent a valuable opportunity to emphasize the distinctive local origin of this niche product, produced from the few remaining pure colonies that could be preserved in western Sicily by only eight beekeepers. Future studies should include larger sample sets to further investigate the hypotheses proposed here regarding the distinctive features of RJ produced by *A. m. siciliana*. In addition, to minimize the risk of admixture with RJ produced by other honeybee subspecies, the samples representative of *A. m. siciliana* were collected exclusively from the islands of Lampedusa, Ustica, and Vulcano. Future studies could extend this investigation to samples collected from mainland Sicily.

## Figures and Tables

**Table 1:** Royal jelly samples used for the study.

Sample	Production Date *	Producer	Production Location	Bee Subspecies
C1	29/01/2024	E.FA	Undeclared	Ligustica
C2	21/11/2023	PR.PH	Undeclared	Ligustica
C3	30/03/2025	E,V	Undeclared	Ligustica
C4	30/06/2024	SEL.	Undeclared	Ligustica
C5	20/11/2024	A.N.	Undeclared	Ligustica
O1	20/05/2024	Ortolani	Emilia-romagna (Ravenna) - Italy	Ligustica
O2	01/07/2024	Ortolani	Emilia-romagna (Ravenna) - Italy	Ligustica
O3	14/03/2024	Petruzzelli	Puglia (Bari) - Italy	Ligustica Hybrid
O4	23/05/2024	Petruzzelli	Puglia (Bari) - Italy	Ligustica Hybrid
O5	16/06/2024	Finelli	Emilia-romagna (Bologna) - Italy	Ligustica
O6	21/07/2024	Finelli	Emilia-romagna (Bologna) - Italy	Ligustica
S1	06/06/2024	Cirrito	Sicily (Palermo) - Italy	*Siciliana* (Ustica island)
S2	02/06/2024	Cirrito	Sicily (Palermo) - Italy	*Siciliana* (Ustica island)
S3	02/06/2024	Buccheri	Sicily (Palermo) - Italy	*Siciliana* (Vulcano island)
S4	05/06/2024	Buccheri	Sicily (Palermo) - Italy	*Siciliana* (Vulcano island)
S5	16/07/2024	Sapienza	Sicily (Palermo) - Italy	*Siciliana* (Lampedusa isl.)

* Based on expiry date and considering 18 months of shelf life.

**Table 2:** Molecules significantly different between commercial samples (C) and artisanal samples from *siciliana* (S) honeybee subspecies.

	C	S	Trend (S-C)	*p*
Choline	1.13 × 10^−1^ ± 3.54 × 10^−2^	5.85 × 10^−2^ ± 1.73 × 10^−2^	↓	<0.05
Glucose	4.74 ± 0.84	3.57 ± 0.60	↓	<0.05
Guanosine	1.08 × 10^−2^ ± 2.88 × 10^−3^	2.44 × 10^−2^ ± 7.35 × 10^−3^	↑	<0.01
1Melibiose	8.04 × 10^−2^ ± 1.23 × 10^−2^	5.94 × 10^−2^ ± 6.12 × 10^−3^	↓	<0.05
Pantothenate	1.87 × 10^−3^ ± 7.79 × 10^−4^	5.70 × 10^−3^ ± 1.65 × 10^−3^	↑	<0.01
Succinate	3.11 × 10^−3^ ± 5.69 × 10^−4^	5.09 × 10^−3^ ± 1.81 × 10^−3^	↑	<0.01
UDP-glucose	4.37 × 10^−2^ ± 4.44 × 10^−3^	2.96 × 10^−2^ ± 8.45 × 10^−3^	↓	<0.05

**Table 3:** Molecules significantly different between artisanal samples from *ligustica* (O) and *siciliana* (S) honeybee subspecies.

	O	S	Trend (S-O)	*p*
10-Hydroxy-2E-decenoate	1.37 ± 0.22	0.77 ± 0.23	↓	<0.01
4-Hydroxybenzoate	1.74 × 10^−2^ ± 3.03 × 10^−3^	7.77 × 10^−3^ ± 2.96 × 10^−3^	↓	<0.01
Adenosine	4.03 × 10^−2^ ± 5.90 × 10^−3^	8.57 × 10^−2^ ± 4.23 × 10^−2^	↑	<0.01
Cellobiose	6.38 × 10^−2^ ± 4.70 × 10^−3^	3.95 × 10^−2^ ± 1.27 × 10^−2^	↓	<0.01
Choline	3.64 × 10^−2^ ± 3.56 × 10^−3^	5.85 × 10^−2^ ± 1.73 × 10^−2^	↑	<0.01
Guanosine	7.39 × 10^−3^ ± 2.95 × 10^−3^	2.44 × 10^−2^ ± 7.35 × 10^−3^	↑	<0.01
Sebacate	3.47 × 10^−1^ ± 5.35 × 10^−2^	1.95 × 10^−1^ ± 6.93 × 10^−2^	↓	<0.01
UDP-glucose	4.14 × 10^−2^ ± 2.95 × 10^−3^	2.96 × 10^−2^ ± 8.45 × 10^−3^	↓	<0.01
Uridine	7.61 × 10^−3^ ± 2.50 × 10^−3^	1.52 × 10^−2^ ± 8.68 × 10^−3^	↑	<0.05
Valerate	1.04 × 10^−2^ ± 1.81 × 10^−3^	5.41 × 10^−3^ ± 1.84 × 10^−3^	↓	<0.01

## Data Availability

The original contributions presented in this study are included in the article/Supplementary Materials. Further inquiries can be directed to the corresponding author.

## Supplementary Materials

The following supporting information can be downloaded at https://www.mdpi.com/article/10.3390/foods15173101/s1, Figure S1: Concentration of the molecules significantly different between samples C and S; Table S1: *p*-values of the comparisons between commercial samples (C) and artisanal samples from siciliana (S) honeybee’s subspecies, reported simplified in Table 2; Table S2: *p*-values of the comparisons between artisanal samples from ligustica (O) and siciliana (S) honeybees subspecies, reported simplified in Table 3.
