# Supplementary material for: Exploratory Characterization of Artisanal Royal Jelly Produced by Apis mellifera siciliana Using 1H-NMR Metabolomics and NIR Spectral Fingerprinting
Source: Foods. 2026 Sep 1;15(17):3101. doi: 10.3390/foods15173101 (PMC13564946; doi:10.3390/foods15173101)
Supplement: Supplementary file 1 [file foods-15-03101-s001.zip › foods-4482630-supplementary.pdf]

Exploratory Characterization of Artisanal Royal Jelly Produced by Apis mellifera siciliana Using 1H-NMR Metabolomics and NIR Spectral Fingerprinting

Supplemental material

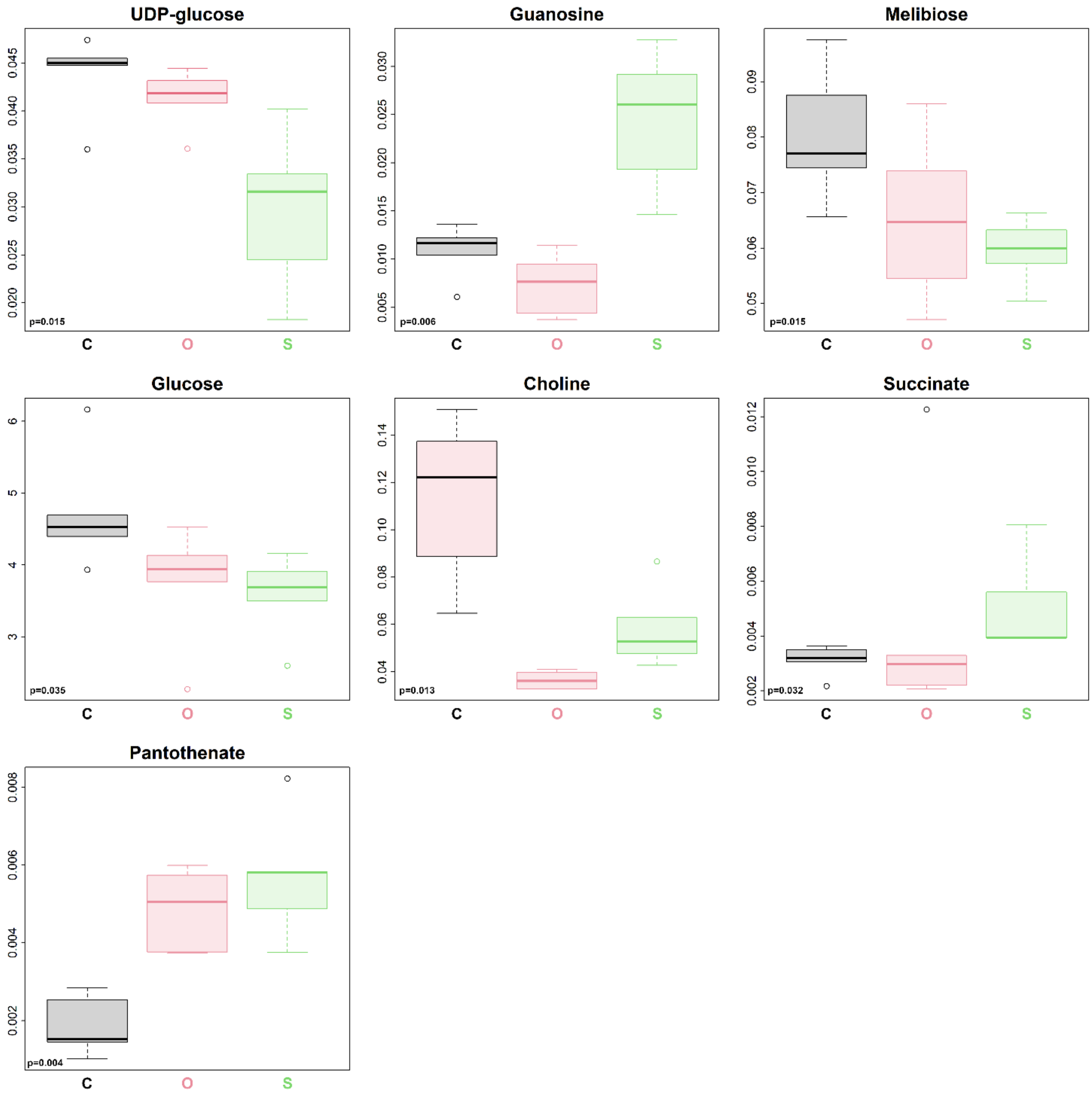

Figure S1. Concentration of the molecules significantly different between samples C and S.

Table S1. p-values of the comparisons between commercial samples (C) and artisanal samples from *siciliana* (S) honeybee's subspecies, reported simplified in Table 2.

	P	*P* adjusted*
Choline	$4.33x10^{-3}$	$8.39x10^{-2}$
Glucose	$4.33x10^{-3}$	$8.39x10^{-2}$
Guanosine	$1.73x10^{-2}$	$8.39x10^{-2}$
Melibiose	$4.33x10^{-3}$	$8.39x10^{-2}$
Pantothenate	$4.33x10^{-3}$	$8.39x10^{-2}$
Succinate	$4.33x10^{-3}$	$8.39x10^{-2}$
UDP-glucose	$4.33x10^{-3}$	$8.39x10^{-2}$

*Benjamini–Hochberg FDR-adjusted p-values

Table S2. p-values of the comparisons between artisanal samples from *ligustica* (O) and *siciliana* (S) honeybee's subspecies, reported simplified in Table 3.

	p	*P* adjusted*
10-Hydroxy-2E-decenoate	$4.33x10^{-3}$	$3.12x10^{-2}$
4-Hydroxybenzoate	$4.33x10^{-3}$	$3.12x10^{-2}$
Adenosine	$8.66x10^{-3}$	$3.46x10^{-2}$
Cellobiose	$4.33x10^{-3}$	$3.12x10^{-2}$
Choline	$4.33x10^{-3}$	$3.12x10^{-2}$
Guanosine	$4.33x10^{-3}$	$3.12x10^{-2}$
Sebacate	$8.66x10^{-3}$	$3.46x10^{-2}$
UDP-glucose	$8.66x10^{-3}$	$3.46x10^{-2}$
Uridine	$3.03x10^{-2}$	$1.09x10^{-1}$
Valerate	$8.66x10^{-3}$	$3.46x10^{-2}$

*Benjamini–Hochberg FDR-adjusted p-values
